# Efficiency of N_2_ Gas Flushing Compared to the Lactoperoxidase System at Controlling Bacterial Growth in Bovine Raw Milk Stored at Mild Temperatures

**DOI:** 10.3389/fmicb.2016.00839

**Published:** 2016-06-02

**Authors:** Patricia Munsch-Alatossava, Romanie Quintyn, Ingrid De Man, Tapani Alatossava, Jean-Pierrre Gauchi

**Affiliations:** ^1^Department of Food and Environmental Sciences, University of HelsinkiHelsinki, Finland; ^2^Food Technology, Vives University College Campus RoeselareRoeselare, Belgium; ^3^MaIAGE, INRA, Université Paris-SaclayJouy-en-Josas, France

**Keywords:** raw milk, N_2_ gas, lactoperoxidase system, antibacterial, Ryan-Einot-Gabriel-Welsch test

## Abstract

To prevent excessive bacterial growth in raw milk, the FAO recommends two options: either cold storage or activation of the lactoperoxidase system (LPs/HT) in milk with the addition of two chemical preservatives, hydrogen peroxide (H) and thiocyanate (T). N_2_ gas flushing of raw milk has shown great potential to control bacterial growth in a temperature range of 6–12°C without promoting undesired side effects. Here, the effect of N_2_ gas (N) was tested as a single treatment and in combination with the lactoperoxidase system (NHT) on seven raw milk samples stored at 15 or 25°C. For the ratio defined as bacterial counts from a certain treatment/counts on the corresponding control, a classical Analyse of Variance (ANOVA) was performed, followed by mean comparison with the Ryan-Einot-Gabriel-Welsch multiple range test (REGWQ). Altogether, the growth inhibition was slightly but significantly higher at 25°C than at 15°C. Except for one sample, all ratios were lower for HT than for N alone; however, these differences were not judged to be significant for five samples by the REGWQ test; in the remaining two samples, N was more effective than HT in one case and less effective in the other case. This study shows that N_2_ gas flushing, which inhibited bacterial growth in raw milk at 15 and 25°C for 24 and 12 h, respectively, could constitute an alternative to LPs where no cold storage facilities exist, especially as a replacement for adulterating substances.

## Introduction

Despite high-tech food production systems, in so-called developed countries, approximately 30–40% of food is lost during retail, in food service or at home; the losses equal those from developing countries, which are mostly attributable to the absence of food chain infrastructure (Gustavsson et al., 2011). In Europe, as much as 20% of the dairy products are lost or wasted every year (FAO, 2012). The presence of spoilage microorganisms, antibiotic-resistant bacteria and the risk of food-borne pathogens are a great challenge to food production systems. Although, milk is considered to be sterile when secreted from a healthy udder, numerous contamination sources increase its bacterial load; hence, depending on the production area, the farm type and the milk handling practices, the types and levels of bacteria vary greatly (Chambers, 2002; Frank and Hassan, 2002). Natural bacterial inhibitors present in raw milk, like immunoglobulins, lactoferrin, and lactoperoxidase prevent bacterial growth for the first 3–4 h (following milking) at ambient temperatures (Korhonen and Marnila, 2002; Marnila and Korhonen, 2002; Ur-Rehman and Farkye, 2002; FAO, 2005); subsequently, the rich nutrient content of milk makes it an ideal growth medium for various bacterial types.

The Codex Alimentarius/FAO recommends two methods for preserving raw milk. Cooling to about 4°C is expected to maintain the original quality of raw milk until processing and consumption; however, within a couple of days, so-called psychrotrophic bacteria take over and promote spoilage of the raw milk due to the production of enzymes (proteases, lipases, phospholipases). Because many of these enzymes withstand the classical heat treatments to which raw milk is subjected, the spoilage action is prolonged until the final products (Chambers, 2002). Where economical or technical constraints prevent the use of cooling facilities, activation of the lactoperoxidase system (LPs) is recommended (FAO, 1991, 2005). However, in certain countries, as the demand for milk and its production have increased, milk adulteration cases are more frequently recorded (Botelho et al., 2015). Around the world, the list of adulterating substances used to preserve milk from microbial contamination or from excessive microbial growth comprises formaldehyde (formalin), soap, detergents, sodium hydroxide, benzoic acid and antibiotics.

Naturally present in bovine milk, the lactoperoxidase (LP) enzyme, which constitutes the 2nd most abundant enzyme, is an oxidoreductase belonging to the peroxidase family (Ur-Rehman and Farkye, 2002). The potential of LPs to inhibit bacterial growth in milk was already recognized by Hanssen in 1924. The use of H_2_O_2_ as a germicide, to preserve raw milk, was tested as early as 1883 by Schrodt. The activity of the LPs system relies on the interactions of three components: the LP enzyme activated by the addition of thiocyanate ion (SCN^−^) and hydrogen peroxide (H_2_O_2_); both of the latter components are present in raw milk at varying concentrations depending on the species, the breed and the animal's diet. The LPs proves to be both bacteriostatic and bactericidal for a variety of Gram-positive and Gram-negative bacteria present in raw milk; the degree of resistance to LPs is bacterial-type dependent; in addition, incubation conditions like aerobiosis/anaerobiosis and the growth phase stage render bacteria more or less susceptible to LPs; Gram-negative catalase-positive bacteria, such as *Pseudomonas* spp., coliforms may be killed if H_2_O_2_ is supplied exogenously; Gram-positive catalase-negative bacteria, like streptococci or lactobacilli are generally inhibited, but not killed by the LPs (Reiter and Härnulv, 1984; Wolfson and Sumner, 1993; Ur-Rehman and Farkye, 2002; FAO, 2005; Seifu et al., 2005; Fweja et al., 2008). Bafort et al. (2014) confirmed earlier observations that altogether the activity of the LPs appears to be more bacteriostatic than bactericidal. The recommended method for preserving raw milk consists of reactivating the LPs by adding around 10 ppm SCN^−^ and 10 ppm H_2_O_2_ (FAO, 1991, 2005); the shelf life of raw milk can then be extended for 7–8 h under tropical conditions. The inhibitory effect of the treatment strongly depends on the storage temperature of the LPs treated milk: the extension of the keeping quality is 4–7, 7–8, 11–12, 16–17, 24–26, and 5–6 days at 31/35, 30, 25, 20, 15, and 4°C, respectively, and is described to be largely dependent on the initial bacterial load (FAO, 2005).

The limitations of raw milk cold storage, together with the observation that isolates retrieved from raw milk (which apparently spent a longer time in cold storage) have real spoilage potential and more frequently exhibit antibiotic resistance, have motivated research efforts to control bacterial growth in raw milk more effectively (Munsch-Alatossava and Alatossava, 2007; Munsch-Alatossava et al., 2012a,b). Two studies examined the use of N_2_ gas applied in a “closed system” to prevent bacterial growth in raw milk (Murray et al., 1983; Dechemi et al., 2005). By considering an “open system” somehow more realistic, culture-dependent investigations and recent DNA barcoding studies showed that no pathogen, no spoilage bacteria nor any anaerobe was clearly advantaged by applying N_2_ gas flushing treatment to raw milk, despite the fact that 10^4^-fold bacterial counts differentiated N_2_ flushed from non-flushed cold stored milk: under the treatments, mesophiles, psychrotrophs, protease and lipase producers were inhibited, whereas phospholipase producers and *Bacillus cereus*-types were “excluded” during cold storage at 6 or 7°C; anaerobes, lactic acid bacteria and enterobacteria were not favored; pilot plant studies also confirmed the potential of N_2_ gas flushing to control bacterial growth (Munsch-Alatossava et al., 2010a,b, 2011; Gschwendtner et al., 2016). Because the N_2_ gas flushing showed that bacterial growth in raw milk could be kept below 5.0 log-units for 3 days at 12°C (Munsch-Alatossava et al., 2010a), we undertook to determine whether N_2_ gas flushing could be effective at temperatures above 12°C to prevent bacterial growth in raw milk and to compare any effect on raw milk to the well-known antibacterial effects of the LPs. Consequently, seven raw milk samples were stored at either 15 or 25°C: “total bacteria,” Gram-negative bacteria, lactic acid bacteria and lactic streptococci were enumerated on relevant agar types from the milk samples subjected to the activated LPs or to N_2_ gas flushing applied as single or combined treatments.

## Materials and methods

### Materials and treatment of bovine raw milk samples

Seven bovine raw milk samples (M1–M7), representing comingled lorry milk delivered to Helsinki Dairy Ltd in Helsinki (Finland) in April, May and June 2015, were considered for the analyses. The source of H_2_O_2_ was a 30% H_2_O_2_ solution (Perdrogen ^R^ 30 Gew%, Riedel de Häen, Seelze, Germany). The thiocyanate anion SCN^−^ was in the form of NaSCN (Sigma-Aldrich, Steinheim, Germany); a 1% (w/v) stock solution was sterile filtered and cold stored until use. Hydrogen peroxide (H), thiocyanate (T), or both components (HT) were added at 10 ppm each (FAO, 2005). The N_2_ gas (AKA Ltd, Riihimäki, Finland) was 99.999% pure; the flow rate for the continuous N_2_ gas flushing treatments (N) was adjusted to 120 ml/min (Munsch-Alatossava et al., 2010a). All raw milk samples (100 ml per bottle and treatment) were continuously mixed and stored at 15 or 25°C ± 0.1 in a water bath until subjected to analyses. The description of the conditions (applied to every sample) is as follows: C = non treated milk (accounts for the control); T = SCN^−^; H = H_2_O_2_; HT = H_2_O_2_ + SCN^−^; N = N_2_ flushing; NT = N_2_ flushing + SCN^−^; NH = N_2_ flushing + H_2_O_2_; NHT = N_2_ flushing + H_2_O_2_ + SCN^−^.

### Microbiological analyses

For the analyses, at the given sampling points, the raw milk was serially diluted in 0.85% NaCl solution. “Total bacterial counts,” Gram-negative bacteria, lactobacilli and streptococci/lactococci were determined mostly from triplicate (if not duplicate) platings on Plate Count Agar (PCA, LabM, Ltd, Lancashire, UK), MacConkey agar (MC, Becton Dickinson, Le Pont de Claix, France), MRS agar (LabM, Ltd, Lancashire, UK), and M17 agar (Sigma-Aldrich, Steinheim, Germany), respectively, after incubation at 30°C for 3 days under aerobic conditions for PCA and MC plates or at 37°C for 72 h for MRS and M17 plates under anaerobiosis. Anaerobic culture jars and AnaeroGen^TM^ sachets (Oxoid Ltd, Basingstoke, UK) were used for creating the anaerobic incubation conditions.

### Statistical analyses

To compare the efficiency of the different treatments, the analyses considered the ratio defined as “the counts obtained on a certain treatment divided by the counts enumerated on the corresponding control.” The statistical analyses determined the significance of the effects of the three considered factors (“time”/duration of the treatment, “media type” and “condition”/applied treatment); moreover, two-factor and three-factor interactions on the ratios were evaluated. At first, a classical ANOVA (Analysis of Variance) was performed. Considering the *p*-values associated with the effects and interactions (*p* < 0.001) and because the effect of the factor “condition” was always found to be highly significant, a comparison of the ratio means that reflect the response to the seven treatments (T, H, HT, N, NT, NH, and NHT) was then undertaken with the Ryan-Einot-Gabriel-Welsch test (REGWQ) for multiple comparison of means, as described by Hsu (1996), with an alpha risk of 0.05. All calculations were performed with the SAS/STAT software version 9.4/ GLM procedure (SAS Institute, NC, USA).

## Results

### Ranking of the treatments

#### Comparison of raw milk samples

The ANOVA revealed a strong significant “sample effect” indicating that the mean ratios in response, to the seven treatments were significantly different (data not shown). Subsequently, the Ryan-Einot-Gabriel-Welsch (REGWQ) multiple range test, applied to the ratios, led to a significant grouping of the seven raw milk samples (M1–M7) into three categories, depending on the combined inhibitory effects caused by the LPs- and N_2_-based treatments (Table 1). The treatments induced the most contrasting effects for the three samples considered in April as the inhibition of bacterial growth was highest in samples M2 and M4 and lowest for M1.

**Table 1:** **REGWQ ranking of the seven bovine raw milk samples stored at 15 and 25°C and treated with LPs and N_2_ gas**.

**Raw milk sample/Applied storage temperature/Start of the analysis**	**Mean ratio^a^**	**Significance^b^**
M1/15°C/Apr 13, 2015	0.7795	A
M3/15°C/Jun 10, 2015	0.5728	B
M6/25°C/May 25, 2015	0.5324	B
M7/25°C/Jun 8, 2015	0.5030	B
M5/25°C/May 4, 2015	0.4251	B C
M4/25°C/Apr 27, 2015	0.2812	C
M2/15°C/Apr 20, 2015	0.2518	C

a *Mean of the ratios from the seven different examined conditions*.

b *Means with the same letter are not significantly different (alpha risk = 0.05)*.

The four samples stored at 25°C followed each other in the ranking. The efficiency of the treatments to trigger bacterial inhibition appeared to be more dependent on the sample than on the applied temperature as both the highest and lowest inhibitory effects were recorded for two samples (M1 and M2) stored at 15°C (Table 1). The treatments showed a rather equivalent inhibitory effect, -with a difference of 0.0294- for the tightly ranked samples M4 and M2, although they were stored at 25 and 15°C, respectively, which equaled the difference recorded for samples M6 and M7, both as tightly ranked and stored at 25°C (Table 1). Altogether, the treatments proved to be slightly but significantly more inhibitory at 25°C than at 15°C.

#### Raw milk storage at 15°C

The REGWQ test distinguished four categories among the tested conditions depending on their impact on the three milk samples (M1, M2, M3) that were stored at 15°C (Table 2). The sample M2, which presented the highest initial “total counts” (4 log-units) and for which the ratio “counts on MacConkey agar/total counts” was lowest, showed the highest sensitivity to the treatments as the ratios were below 0.15 for five conditions; in contrast, sample M3 presented the lowest initial counts (2.6 log-units) but less inhibition was observed based on the ratios (Table 2). Based on counts from all four agar medium types, condition T was ranked apart in all three experiments with two ratios exceeding 1, indicating that the addition of SCN^−^ exerted a clear stimulatory effect on bacterial growth for samples M1 and M3 (Table 2). The addition of H_2_O_2_ alone (H) triggered an inhibition of approximately 30% for M1 and M3 and of 62% for M2 (Table 2). The activated lactoperoxidase system (HT) induced variable levels of inhibition: 18, 95, and 67% for M1, M2, and M3, respectively (Table 2). For N_2_ flushing (N), the recorded inhibitory percentages were 62, 86, and 38% for M1, M2, and M3, respectively. The difference between N and HT was significant for M1, where N alone supplanted HT; but HT and N were equally efficient for M2 and M3. In all three experiments, the ratio was significantly lower for NT than for T; it was different from N for M1, and similar for M2 and M3, suggesting that the inhibitory effects of N_2_ gas flushing overcame the stimulatory effect of T (Table 2); for all three experiments, the effects due to the combination of N and H (NH) did not differ significantly from those observed for N alone. Compared to H alone, the addition of N to H increased the inhibitory effect for M2 (Table 2). For both milk samples M2 and M3, the inhibitory effect was maximal for condition NHT but not significantly different from condition HT (for M2 and M3) or from N (for M2).

**Table 2:** **Results of the Ryan-Einot-Gabriel-Welsch multiple range test for the three raw milk samples stored at 15°C**.

**Raw milk sample**	**Initial “total counts^a^”**	**Initial counts on MacConkey agar/“total counts”**	**Condition**	**Ratio**	**Significance^b^**	***F*-value of the most important factors**
M1	3.9	ND^c^	T	1.7033	A	Time 32.44
			NT	1.0329	B	Condition 31.02
			HT	0.8197	B C	Medium 27.12
			H	0.7029	C D	
			NHT	0.4540	D	
			N	0.3817	D	
			NH	0.3650	D	
M2	4.0	0.9	T	0.9243	A	Condition 112.23
			H	0.3832	B	Medium 35.15
			NT	0.1437	C	
			N	0.1390	C D	
			NH	0.0547	C D	
			HT	0.0488	C D	
			NHT	0.0127	D	
M3	2.6	1.1	T	1.2316	A	Time 28.02
			H	0.6821	B	Condition 16.84
			N	0.6243	B C	Medium 11.75
			NH	0.5148	B C	
			NT	0.4590	B C D	
			HT	0.3337	C D	
			NHT	0.1716	D	

a “Total counts” on PCA,

b Mean ratios with the same letter are not significantly different (alpha risk = 0.05),

c *ND = not determined*.

#### Raw milk storage at 25°C

The REGWQ test revealed that the treatments were less discriminatory at 25°C, as only three categories were distinguished among the applied treatments for the four milk samples. However, despite greater homogeneity in initial “total counts” (which ranged between 3.1 and 3.8 log-units), the four samples responded differently to the treatments (Table 3).

**Table 3:** **Results of the Ryan-Einot-Gabriel-Welsch multiple range test for the four raw milk samples stored at 25°C**.

**Raw milk sample**	**Initial “total counts”^a^**	**Initial counts on MacConkey agar/“total counts”**	**Condition**	**Ratio**	**Significance^b^**	***F*-value of the most important factors**
M4	3.6	ND^c^	T	0.9027	A	Condition 40.30
			H	0.6019	B	Medium 9.19
			N	0.1823	B C	
			NT	0.1036	C	
			NH	0.0572	C	
			HT	0.0521	C	
			NHT	0.0398	C	
M5	3.2	0.8	H	1.0630	A	Condition 46.01
			T	0.8939	A B	Time 27.87
			NT	0.6955	B	Medium 7.74
			N	0.1733	C	
			NH	0.1060	C	
			HT	0.0271	C	
			NHT	0.0130	C	
M6	3.8	0.9	T	1.2494	A	Condition 23.93
			H	1.1004	A	Time 14.33
			NH	0.3955	B	
			NT	0.3750	B	
			N	0.3324	B	
			NHT	0.1311	B	
			HT	0.1185	B	
M 7	3.1	1.2	T	1.2041	A	Time 26.19
			H	0.5957	B	Condition 22.64
			N	0.5279	B	Medium 12.56
			NH	0.4186	B C	
			NT	0.3541	B C	
			NHT	0.2172	C	
			HT	0.1954	C	

a Total counts” on PCA,

b Mean ratios with the same letter are not significantly different (alpha risk = 0.05),

c *ND = not determined*.

For raw milk samples stored at 25°C, T also stimulated bacterial growth as the mean ratio was highest for M4 and it exceeded 1 for M5, M6, and M7 (Table 3). Condition H yielded ratios undistinguishable from T for M5 and M6, but it showed a moderate inhibitory effect for M4 and M7 (Table 3). HT promoted the highest growth inhibition for samples M6 and M7. If higher inhibitory ratios were recorded for HT compared to N_2_ flushing (N), these differences were not judged to be significant for three samples. At 25°C, with the exception of M7, N_2_ flushing alone (N) achieved a reduction in bacterial levels similar to that obtained with HT (Table 3). NHT promoted the highest inhibition of bacterial growth for samples M4 and M5. Altogether, NHT was tightly ranked with HT as the differences between the two conditions were not significant for any of the investigated raw milk samples (Table 3).

### Contribution of the major factors that determined the ranking

**Figure 1:**
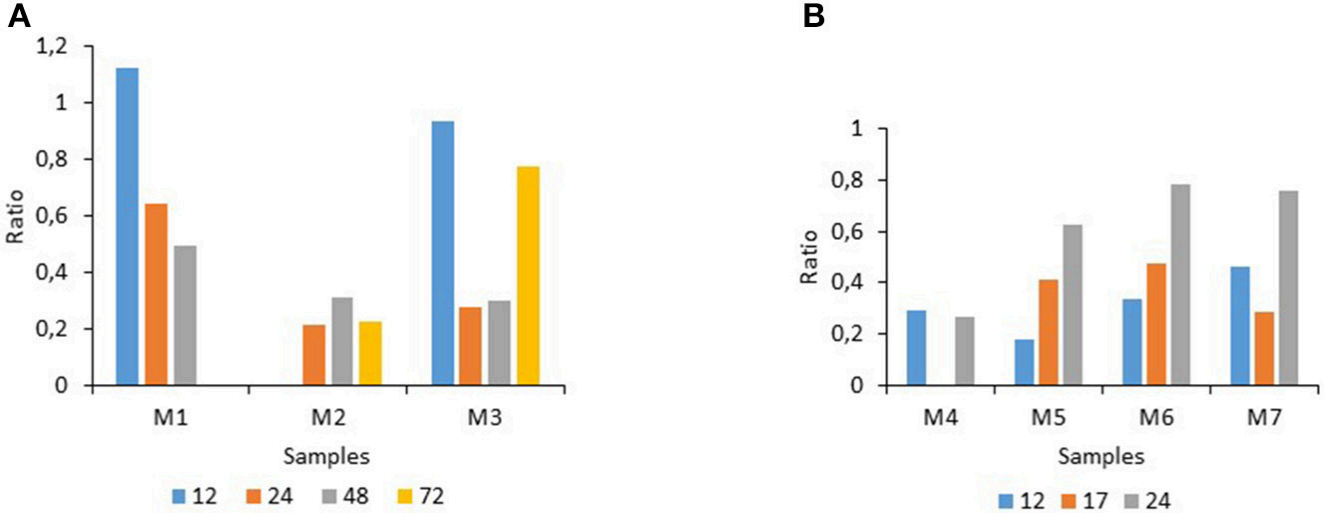
**Impact of the factor “storage time in h” on the ratios determined for raw milk stored at 15°C (A) (for 12–72 h), or at 25°C (B) (for 12–24 h)**.

The analysis of the contribution of the different factors to the ranking of the ratios revealed that the “condition” (or applied treatment), the storage “time” of the milk and the “medium type” affected the seven raw milk samples (M1–M7) differently (Tables 2, 3). Altogether, although the factors varied in their respective importance, the “condition” was a major determinant for four samples (M2, M4, M5, and M6), with an overwhelming impact on the ranking of the treatments for sample M2. Clearly, the factor storage “time” also displayed great heterogeneity among the seven raw milk samples as it mainly determined the ranking of the treatments for samples M3 and M7; altogether, its effect was variable and more moderate as noticeable for M1, for example, where the three factors played an equivalent role (Tables 2, 3). Considering the factor storage “time” during the course of the experiments, at 15°C the inhibitory effects increased during storage for M1, they were high and constant for M2, and they fluctuated over time for M3 being maximal at 24 and 48 h storage (Figure 1A). At 25°C, for samples M5 and M6, the inhibition was maximal at 12 h, then the mean ratios increased over time, indicating declining inhibition; for M7, the highest inhibition occurred at intermediate storage time (17 h), whereas for M4 the inhibition remained unchanged for 24 h. (Figure 1B).

**Figure 2:**
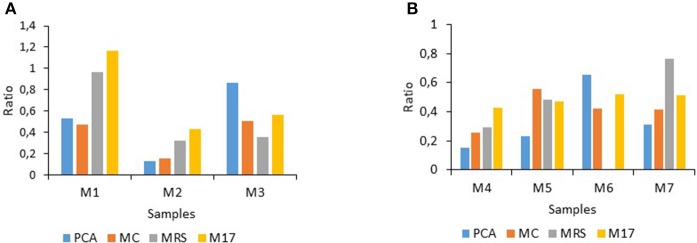
**Ratio levels illustrating the incidence of the treatments on bacterial population types enumerated on four considered agar types for raw milk stored at 15°C (A) or at 25°C (B)**. PCA, (Plate Count Agar); MC, (MacConkey agar); MRS, (culture of lactic acid bacteria); M17, (lactic streptococci).

Consideration of the factor “medium type” (which supported the growth of different bacterial populations, together with the corresponding incubation conditions) showed that altogether the different bacterial population types were not similarly affected by the treatments at either considered storage temperature (Figure 2). For M1, bacterial types on MRS and M17 agar were recovered in high amounts under the treatments compared to PCA and MC whereas for M2, clearly all population types were hindered but mainly those enumerated on PCA and on MC, in contrast to M3 where populations on MRS were mostly inhibited (Figure 2A). At 25°C, the inhibitory effects recorded on the different agar types also showed milk-sample dependent responses. “Total counts” on PCA were inhibited by 85% for M4 but only by 35% for M6. Gram-negative bacterial types were also mostly inhibited for M4 (75%), whereas the inhibition ranged from 60% (M6 and M7) to 45% for M5. The inhibition of lactobacilli varied greatly between 70% (M4) and 25% (M7), in contrast to streptococci/lactococci for which the inhibitory effects (which ranged between 42 and 52%) were mostly similar between all four samples (Figure 2B).

ANOVA tables revealed double and triple interactions between the considered factors storage “time,” “medium” type, and “condition,” which were often significant (at an alpha risk factor of 5%) but generally lower than the main effects due to the three factors alone (data not shown).

### Trends of bacterial growth for samples M2 and M5

**Figure 3:**
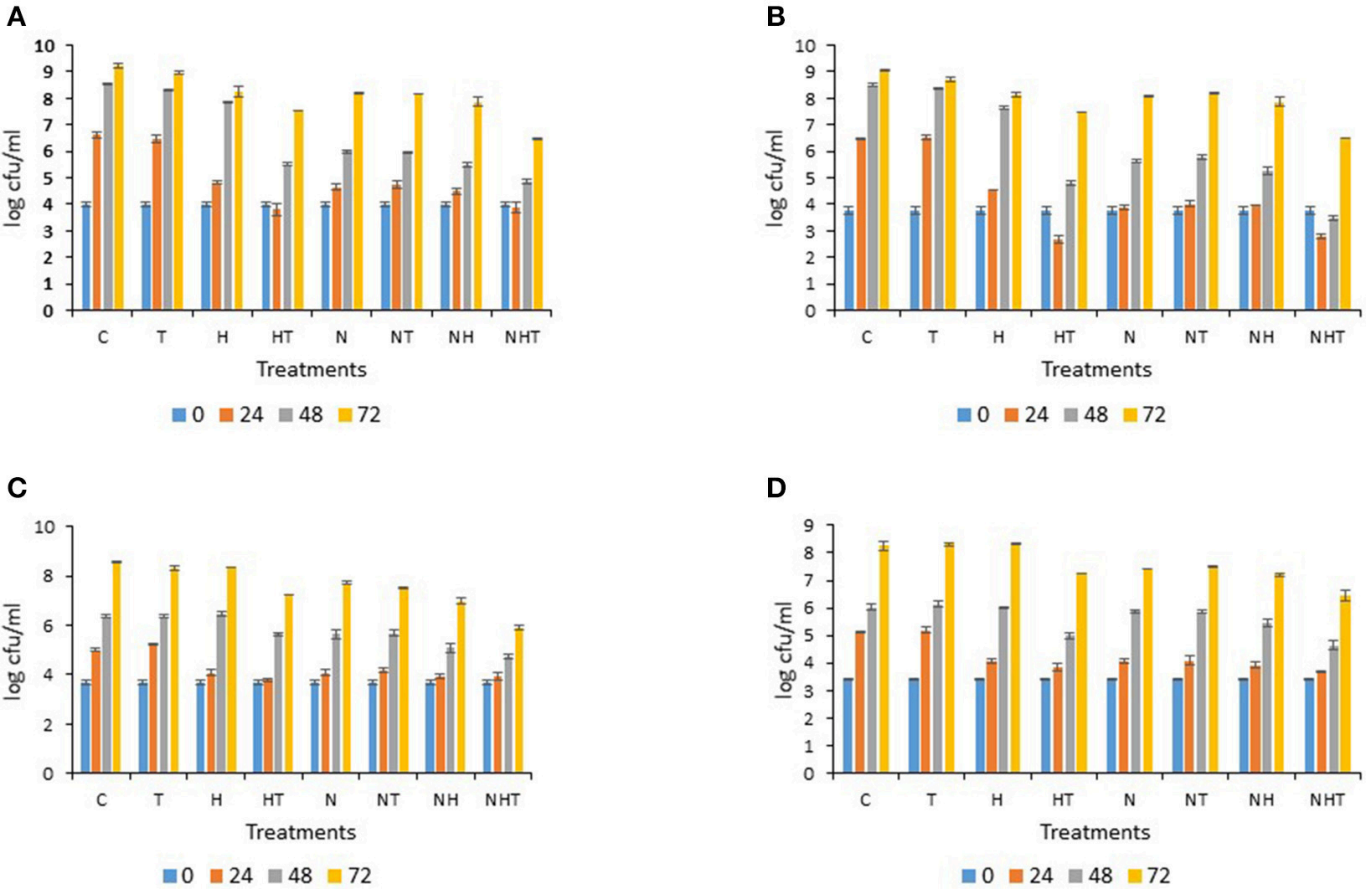
**Bacterial counts determined for sample M2 stored at 15°C on (A) PCA (Plate Count Agar), (B) MacConkey agar, (C) MRS agar, (D) M17 agar: error bars indicate standard deviations based mainly on three replicates**.

**Figure 4:**
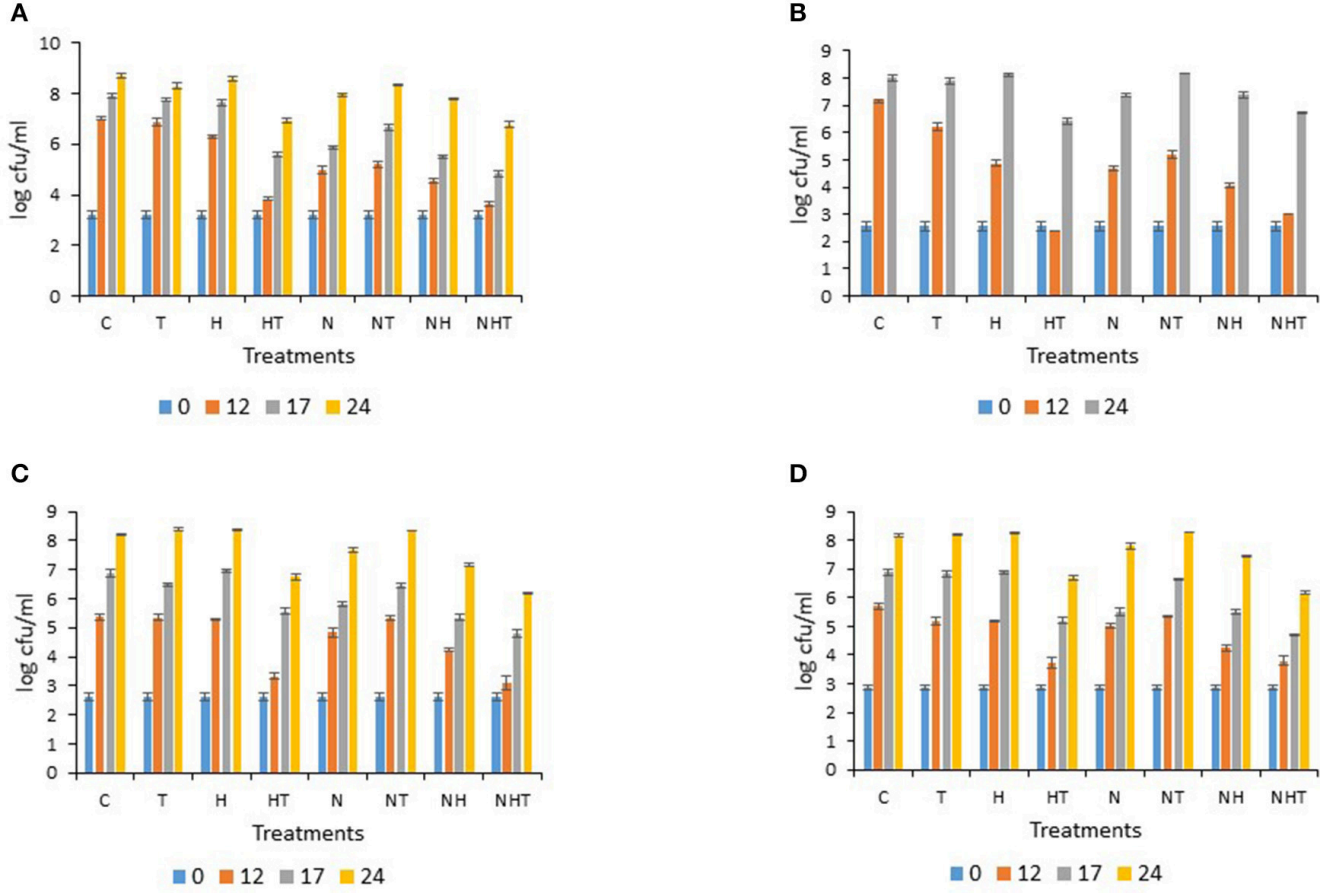
**Bacterial counts determined for sample M5 stored at 25°C on (A) PCA (Plate Count Agar), (B) MacConkey agar, (C) MRS agar, (D) M17 agar: error bars indicate standard deviations based mainly on three replicates**.

The detailed results of the bacterial growth trends for samples M2 and M5, which exhibited high sensitivity to the treatments (Category C in Table 1), are shown in Figures 3, 4. For sample M2 (stored at 15°C), irrespective of the sampling time points, similar bacterial levels were enumerated on PCA for condition T to those of the control C (Figure 3A). Compared to C, at 24 h, inhibition of growth was observed for H (−1.8 log-units), for N (−2.0 log-units), for NT (−1.9 log-units) and for NH (−2.1 log units); conditions HT and NHT yielded lower bacterial levels (−2.8 and −2.7 log-units, respectively) than C. At the 48 and 72 h time points, the “total bacterial counts” for T and H were quite similar to those of C in contrast to all the other conditions (HT, N, NT, NH, and NHT), which showed 1–1.5 log-units less bacteria after 48 h; however, at 72 h, the inhibitory effects faded and only condition NHT showed lower counts (Figure 3A). Of note, like HT, N kept the counts below 5.5 log-units for more than 24 h. For M1 and M3, a similar level of inhibition was observed in one case, and increased inhibition was observed for the other case (data not shown). Over time, on MC agar, C and T showed similar bacterial growth trends (Figure 3B). In contrast, H exhibited counts close to 2 log-units lower than C at 24 h; but the final counts for H were only slightly lower at 48 and 72 h than for C or T. A bactericidal type of action was observed for HT as counts dropped during the first 24 h then increased thereafter. All conditions where milk samples were N_2_-flushed showed that bacterial counts were below 5.5 log-units after 24 h of storage; this observation was still valid at 48 h for NH and NHT, but counts slightly increased for N (5.6 log-units) and NT (5.8 log-units). Notably at 48 and 72 h, the counts were slightly lower for NHT than for HT (Figure 3B). The hindrance of the growth of lactobacilli was only moderate as all conditions showed only slightly lower bacterial levels than C or T at 24 h (Figure 3C). Compared to HT and N, NHT exhibited lower counts after 48 and 72 h. For streptococci/lactococci, all treatments showed approximately 1 log-unit lower counts at 24 h on M17, except for T, which was indistinguishable from C (Figure 3D). At 48 h, all counts had increased similarly except for HT and NHT for which some delay in growth was observed. At 72 h, the counts were approximately 1.8 log-units lower for NHT than for C (Figure 3D).

For M5, after 12 h storage at 25°C, conditions C, H, and T exhibited over 6.5 log-units “total counts” on PCA, whereas all other conditions kept bacterial levels below 5.5 log-units. At 17 h, counts from HT, NH, and NHT were below or approximately at 5.5 log-units, whereas they reached 5.9 log-units for N (Figure 4A). A slight inhibitory effect was still noticeable at 24 h for conditions HT and NHT. On MC, after 12 h storage at 25°C, Gram-negative bacterial counts had increased greatly for C and T; H and N alone or in combination with T (NT) yielded counts that ranged between 4.7 and 5.2 log-units; at 24 h, HT and NHT showed lower bacterial counts (Figure 4B). On MRS, inhibited lactobacilli growth was mainly observed with HT and NHT as counts were lowest at 12, 17, and 24 h (Figure 4C). If HT, in contrast to N inhibited the growth of lactobacilli during the initial 12 h of storage, at 17 h, the level was equivalent for HT and N. On M17, streptococcal/lactococcal counts increased similarly for C, T, H, N, and NT (Figure 4D), in contrast to HT, NH and NHT where counts were lower after 12 h of storage. At 17 h, some inhibitory effect was still noticed for HT, N, NH, and NHT, as it was for HT and NHT at 24 h (Figure 4D).

## Discussion

In this study, N_2_ gas flushing and chemical preservatives (SCN^−^ and H_2_O_2_ which serve to activate the lactoperoxidase system (LPs) in raw milk) were used to treat seven lorry raw milk samples (M1–M7) that presented a “certain” bacterial load and diversity resultant from a “certain” cold storage history; as “total counts” ranged between 2.6 and 4.0 log-units at the initial stage, all seven raw milk samples can be considered of very good bacteriological quality (Tables 2, 3) as “total bacterial counts” per ml in the tank at the farm and at the dairy silo level should not exceed 10^5^ and 3.10^5^ cfu/ml (5.48 log-units), respectively (Anonymous, 2004).

The treatments were limited to 24 h at 25°C and to 72 h at 15°C when also most treated milk samples exceeded 5.5 log-units in “total counts” (Figures 3A, 4A and data not shown). Notably, despite a 10°C difference in the applied storage temperature, a certain similarity between the inhibitory patterns was noticeable for M2 and M4, and for M3 and M6 (with the exception of lactobacilli, not determined) (Figure 2 and data not shown), suggesting that populations could be similarly targeted by the treatments irrespective of the storage temperature.

Altogether, the treatments were most efficient for samples M2, M4, and M5 (Table 1). Analysis of the factors that impacted the ranking of the raw milk samples revealed that for these samples, the factor “condition” was preponderant in the ranking (Tables 2, 3).

Considering the values of the ratios that reflect the inhibition due to the treatments, altogether, the inhibitory effects of either the LPs or the N_2_ gas flushing were more dependent on the sample (or initial microbiota) than the initial bacterial level (Tables 1, 3); this point is well illustrated by the position of sample M2 in the ranking (Table 1) which showed the highest sensitivity to the applied treatments despite the fact that it presented the highest initial bacterial counts. Perhaps this result could be explained by better synergy with the indigenous LPs in this raw milk sample or by the presence of other bacterial inhibitors (Korhonen and Marnila, 2002; Marnila and Korhonen, 2002).

The microbiological analyses showed that bacterial counts on MacConkey agar/“ total counts”on PCA agar were lowest for M2, M5, and M6, and higher for M3 and M7 (Tables 2, 3). Determination of the inhibitory ratios revealed that for treatments HT and N, the values were lowest for samples M2, M5, and M6 and highest for M3 and M7 (Tables 2, 3). It is well known that cold storage of raw milk promotes an increase of Gram-negative bacterial types (Chambers, 2002); altogether, our data suggest that both HT and N treatments achieved the highest inhibition of bacterial growth in milk samples not yet totally dominated by Gram-negative bacteria.

The performance of the activated lactoperoxidase system (HT) enabled bacterial growth to be controlled for 24 h in raw milk stored at 15°C (for M1 until 48 h) (Figure 3A and data not shown), and for 12 h in milk stored at 25°C (Figure 4A and data not shown), respectively, which is in line with previous reports (FAO, 2005). When the milk was stored at 15°C, the N_2_ gas flushing alone was effective at controlling bacterial growth for 24 h (for M2 and M3) and for 48 h (for M1) (Figure 3A, and data not shown); similarly, bacterial growth was halted for 12 h in all four milk samples (M4–M7) stored at 25°C (Figure 4A, and data not shown). Importantly, no bacterial types were clearly stimulated during the N_2_ flushing treatments, at either considered temperature, corroborating previous observations of raw milk stored at 6 and 12°C (Munsch-Alatossava et al., 2010a; Gschwendtner et al., 2016). Compared to HT, the effectiveness of N_2_ for the same milk sample revealed that with one exception (M1 stored at 15°C, Table 2), all ratio values determined for N exceeded the values for HT, suggesting that altogether HT was more effective at preventing bacterial development; however, these differences were not found to be statistically significant with the REGWQ test at either considered temperature (15 or 25°C). Consequently, within a similar time frame, N_2_ can achieve control of bacterial populations equivalent to that of the activated lactoperoxidase system (HT) (Tables 2, 3), although unlike HT, N_2_ did not trigger any detectable bactericidal type of effect considering the original bacterial load (Figures 3B, 4B and data not shown).

As noticeable in Figures 3B, 4B, bacterial counts on MacConkey agar were lower or slightly lower at 24 h at 15°C and 12 h at 25°C than the initial counts for condition HT (this result was also observed for other samples, data not shown), illustrating the bactericidal effect of the LPs toward Gram-negative bacteria described earlier (Wolfson and Sumner, 1993; Ur-Rehman and Farkye, 2002; FAO, 2005; Seifu et al., 2005). Here, the effect of SCN^−^ (T), sometimes clearly stimulatory, was mostly converted into high inhibition of bacterial development with the simultaneous presence of H_2_O_2_ (HT) for six raw milk samples (with the exception of M1) (Tables 2, 3). Here also, the activated LPs (HT) was less effective against lactic acid bacteria, which is in agreement with previous reports (FAO, 2005).

Where refrigeration is available, the addition of H_2_O_2_ is not generally recommended for raw milk; however, its use is permitted in certain applications prior to cheese making or during the preparation of modified whey, in the USA for example (Martin et al., 2014). The addition of H_2_O_2_ alone (H) here did not induce high inhibition with the exception of M2 (Figure 3B). NHT yielded the lowest and the second lowest ratio in 4 and 2 cases, respectively, at 25 and 15°C (Tables 2, 3); these ratio values were very close to those obtained for HT (except for sample M3, where the difference reached 0.16). Considering the performance of HT, no synergistic effect could be induced by the additional application of N_2_ gas flushing to the lactoperoxidase system; however, by comparing the ratios recorded for N with those of NHT, the addition of the LPs system provoked superior inhibition to that of N_2_ flushing alone for six samples (Tables 2, 3), which altogether could suggest that the LPs- based treatment (HT) and the N_2_ gas flushing do not target the exact same bacterial groups in raw milk.

Considering the applied treatments (with exception of T), for all bacterial populations recovered on PCA, MRS, and M17 agar, the effects were mostly bacteriostatic as a growth increase was recorded after some delay (Figures 3A, 4A, and data not shown). At both considered temperatures, a bacteriostatic type of action was also observed here with N_2_ gas flushing (Figures 3, 4 and data not shown), which is in line with results from earlier studies when milk samples were flushed during storage between 6 and 12°C (Murray et al., 1983; Munsch-Alatossava et al., 2010a). No bactericidal type of action was noticed with N in this study, in contrast to HT. However, that N_2_ gas flushing does not exert any bactericidal action is not that straightforward considering recent observations showing that N_2_ gas flushing can act as a bactericidal agent for *Bacillus weihenstephanensis*, a Gram-positive representative found in pasteurized milk (Lechner et al., 1998; Munsch-Alatossava et al., 2013), as a majority of cells were considerably damaged based on electron microscopic observations following the flushing treatment (Munsch-Alatossava and Alatossava, 2014). Most probably, our plating conditions, which did not specifically target *Bacillus* types of Gram-positive bacteria, together with the fact that *Bacillus* is usually present in low numbers in raw milk, prevented the detection of any bactericidal type of action in this study.

HT exerted rather rapid action as after 7 h at 15°C, the bacterial counts were lower than the initial counts on MacConkey agar (Figure 3B). The factor “time” seems to play also a crucial role when considering the mode of action of N_2_ gas: for raw milk samples stored at 6°C, it appeared that phospholipase producers were excluded in a sample-dependent manner under the N_2_ flushing treatment after 3, 7, or 11 d (Munsch-Alatossava et al., 2010b). Additionally, many days were necessary to record a bactericidal type of action on *Bacillus weihenstephanensis* under the same continuous N_2_ gas flushing (Munsch-Alatossava and Alatossava, 2014), which altogether suggests that unlike HT (which exerts rather rapid action), the action of N_2_ gas flushing seems to be inscribed in time.

## Conclusion

N_2_, as a non-finite resource and categorized as GRAS (Generally Regarded As Safe), also allowed in organic production systems, presents several advantages: mainly, it enables the prolonged cold storage of milk by inhibiting spoilage bacteria without favoring well-known human pathogens or other harmful bacteria. This study shows that in the absence of cold chain facilities, N_2_ flushing could be used within a controlled time frame (12 and 24 h at 25 and 15°C, respectively) to control bacterial growth in raw milk. Very likely, the treatment could also compensate for cold chain failures. Further studies are necessary to optimize the effectiveness of the N_2_ gas flushing treatment combining minimal costs with maximal efficiency to offer an alternative to the use of chemicals, especially replacing the use of illegal adulterants with negative impacts on human health and the environment.

## Author contributions

PMA, IDM, and TA conceived and designed the experiments: PMA, RQ performed the experiments; JPG analyzed the data; TA, JPG contributed reagents/materials/analysis tools; PMA, TA, and JPG wrote the paper.

### Conflict of interest statement

The authors declare that the research was conducted in the absence of any commercial or financial relationships that could be construed as a potential conflict of interest.
